# Supplementary material for: Light-modulated neural control of sphincter regulation in the evolution of through-gut
Source: Nat Commun. 2024 Oct 18;15:8881. doi: 10.1038/s41467-024-53203-7 (PMC11489725; doi:10.1038/s41467-024-53203-7)
Supplement: Supplementary file 1 — Supplementary Information [file 41467_2024_53203_MOESM1_ESM.pdf]

Light-modulated neural control of sphincter regulation in the evolution of through-gut.

Junko Yaguchi, Kazumi Sakai, Atsushi Horiuchi, Takashi Yamamoto, Takahiro Yamashita, *Shunsuke Yaguchi

*Corresponding author; Shunsuke Yaguchi, Shimoda Marine Research Center, University of Tsukuba, 5-10-1 Shimoda, Shizuoka, 415-0025 Japan

Phone; +81-558-22-1317

Fax; +81-558-22-0346

E-mail; yag@shimoda.tsukuba.ac.jp

Supplementary Information

Supplementary Figures

Supplementary Figure 1

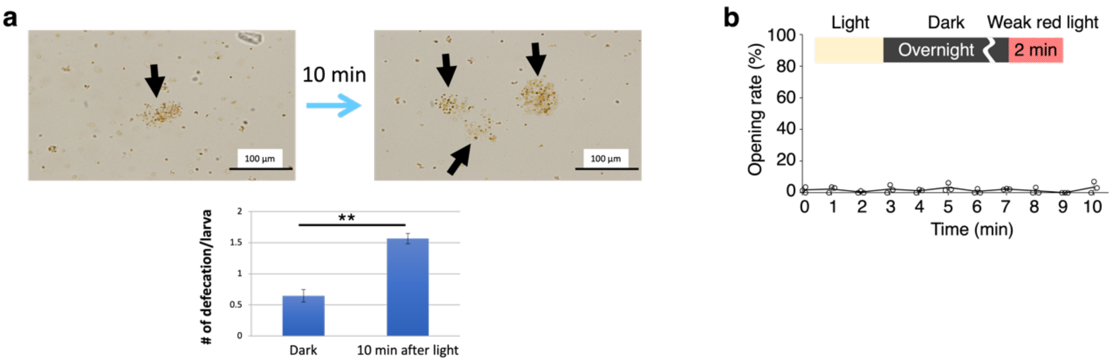

Light-Dependent Defecation Through Anal Opening Excludes Red Light Influence. **a.** Quantitative analysis of excrement counts before and after photoirradiation, highlighting the light-induced defecation response. Arrows indicate visible excrements. The graph shows the average number of defecation per larva after 10 min light transferred from overnight dark condition. The larvae were kept in the dark in a ϕ3.5 cm dish containing 3.0 ml of seawater. We counted the number of excretions in the dish at the moment the light was turned on and again 10 minutes later. The average number of excrement was subdivided with the number of embryos (N = 6 batches [each consisting of a different male and female pair], *n* = 79, 67, 69, 70, 62, 86 larvae). **b.** Observations reveal infrequent anal opening in response to red light, indicating a specific wavelength dependency for light-induced defecation mechanisms (N = 3 batches, *n* [0 min] = 42, 82, 66 larvae, *n* [1 min] = 37, 66, 28 larvae, *n* [2 min] = 37, 82, 60 larvae, *n* [3 min] = 27, 20, 55 larvae, *n* [4 min] = 50, 77, 70 larvae, *n* [5 min] = 48, 32, 61 larvae, *n* [6 min] =36, 79, 67 larvae, *n* [7 min] = 51, 43, 75 larvae, *n* [8 min] = 52, 47, 59 larvae, *n* [9 min] = 57, 43, 45 larvae, *n* [10 min] = 65, 56, 96 larvae). Statistical significance is denoted as $**p < 0.01$, Welch's t test (two-sided). Error bars shown in (a) indicate SEM. Scale bar = 100 µm.

Source data are provided as a Source Data file.

Supplementary Figure 2

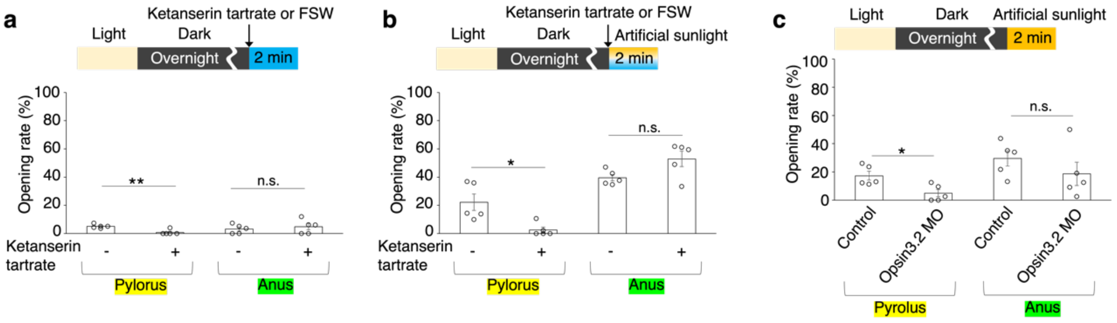

Regulation of Pyloric Opening by the Opsin3.2-Serotonin Pathway. a,b. Comparative analysis of the pyloric and anal opening rates in sea urchin larvae following treatment with the serotonin receptor inhibitor ketanserin tartrate (10 µM), without (**a**) and with (**b**) photoirradiation (N [a] = 5 batches [each consisting of a different male and female pair], *n* [ketanserin tartrate -] = 69, 39, 39, 27, 27 larvae, *n* [ketanserin tartrate +] = 19, 16, 33, 15, 25 larvae; N [b]= 5 batches, *n* [ketanserin tartrate -] = 104, 70, 115, 114, 92 larvae, *n* [ketanserin tartrate +] = 18, 33, 37, 47, 47 larvae). The graphs illustrate the differential impact of light exposure on the serotonin-mediated control of opening behaviors. Although Ketanserin tartrate have potential to inhibit other neurotransmitter pathways[1], the inhibitory effect of the pyloric opening has been shown as same as that in serotonin receptor morphants in previous study[2]. **c.** Opening rates of the pylorus and anus in control larvae versus Opsin3.2 morphants, measured 2 minutes post-photoirradiation, highlighting the role of Opsin3.2 in light-dependent regulation (N=5 batches, *n* [control] = 38, 77, 71, 106, 46 larvae, *n* [Opsin3.2 morphants] = 48, 39, 16, 21, 11 larvae). Statistical significance is indicated as *$p < 0.05$, **$p < 0.01$; n.s. = not significant, Welch's t test (two-sided). Error bars shown in all graphs indicate SEM. Source data are provided as a Source Data file.

Supplementary Figure 3

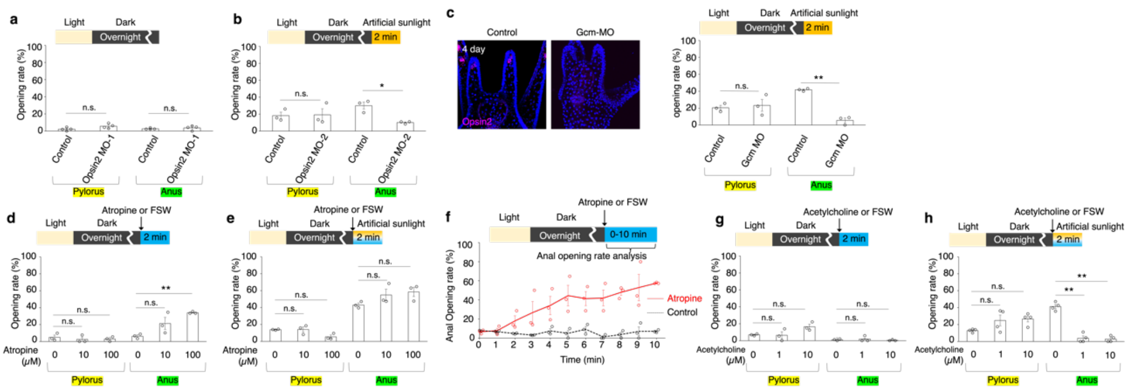

Involvement of the Opsin2 and Cholinergic Pathway in Anal Opening. a. Comparison of pyloric and anal opening rates in control versus Opsin2 morphants without photoirradiation, highlighting the role of Opsin2 in anal opening regulation (N = 4 batches [each consisting of a different male and female pair], *n* [control] = 43, 53, 45, 46 larvae, *n* [Opsin2 MO-1] = 34, 32, 48, 43 larvae). **b.** Opening rates of the pylorus and anus in control and morphants, in which the second Opsin2 morpholino was injected, 2 minutes post-photoirradiation, demonstrating the light-dependent activation of the Opsin2 pathway (N = 3 batches, *n* [control] = 109, 77, 46 larvae, *n* [Opsin2 MO-2] = 82, 37, 12 larvae). **c.** Reduction of Opsin2-expressing cells in Gcm morphants and its effect on opening rates. Opening rates of the pylorus and anus in control versus Gcm morphants are shown 2 minutes after photoirradiation (N = 3 batches, *n* [control] = 30, 44, 43 larvae, *n* [Gcm MO] = 34, 18, 25 larvae). **d,e.** Effects of the muscarinic acetylcholine receptor inhibitor, atropine, on opening rates at various concentrations, observed without (**d**) and with (**e**) photoirradiation, to assess the cholinergic pathway's contribution to opening mechanisms (N [d] = 3 batches, *n* [0 µM] = 57, 118, 61 larvae, *n* [10 µM] = 16, 39, 52 larvae, *n* [100 µM] = 18, 42, 31 larvae; N [e] = 3 batches, *n* [0 µM] = 38, 104, 115 larvae, *n* [10 µM] = 37, 19, 38 larvae, *n* [100 µM] = 34, 21, 27 larvae). **f.** Time-course analysis

of anal opening rates in sea urchins treated with atropine without photoirradiation, elucidating the temporal dynamics of cholinergic inhibition (N [control] = 3 batches, *n* [0 min] = 49, 55, 25 larvae, *n* [1 min] = 39, 20, 28 larvae, *n* [2 min] = 24, 21, 19 larvae, *n* [3 min] = 37, 30, 31 larvae, *n* [4 min] = 33, 25, 23 larvae, *n* [5 min] = 19, 30, 21 larvae, *n* [6 min] =48, 28, 22 larvae, *n* [7 min] = 28, 20, 14 larvae, *n* [8 min] = 25, 27, 13 larvae, *n* [9 min] = 30, 19, 19 larvae, *n* [10 min] = 23, 30, 17 larvae; N [atropine] = 3 batches, *n* [0 min] = 49, 42, 23 larvae, *n* [1 min] = 32, 26, 15 larvae, *n* [2 min] = 36, 28, 26 larvae, *n* [3min] = 36, 31, 16 larvae, *n* [4 min] = 35, 26, 29 larvae, *n* [5 min] = 37, 25, 17 larvae, *n* [6 min] = 36, 21, 26 larvae, *n* [7 min] = 21, 25, 14 larvae, *n* [8 min] = 29, 14, 21 larvae, *n* [9 min] = 43, 29, 15 larvae, *n* [10 min] = 21, 12, 14 larvae). **g,h.** Impact of acetylcholine addition on the opening rates of the pylorus and anus at various concentrations, without (**g**) and with (**h**) photoirradiation, further confirming the cholinergic pathway's involvement in light-mediated opening responses (N [g] = 3 batches, *n* [0 μM] = 50, 87, 101 larvae, *n* [1 μM] = 17, 37, 36 larvae, *n* [10 μM] = 81, 48, 47 larvae; N [h] = 4 batches, *n* [0 μM] = 38, 104, 70, 115 larvae, *n* [1 μM] = 46, 23, 40, 36 larvae, *n* [10 μM] = 30, 14, 49, 46 larvae). We used one-way ANOVA followed by Tukey's post hoc test in (**d**)(**e**)(**g**)(**h**). Statistical significance is denoted as $*p < 0.05$, $**p < 0.01$; n.s. = not significant, Welch's t test (two-sided). Error bars shown in all graphs indicate SEM. Source data are provided as a Source Data file.

Supplementary Figure 4

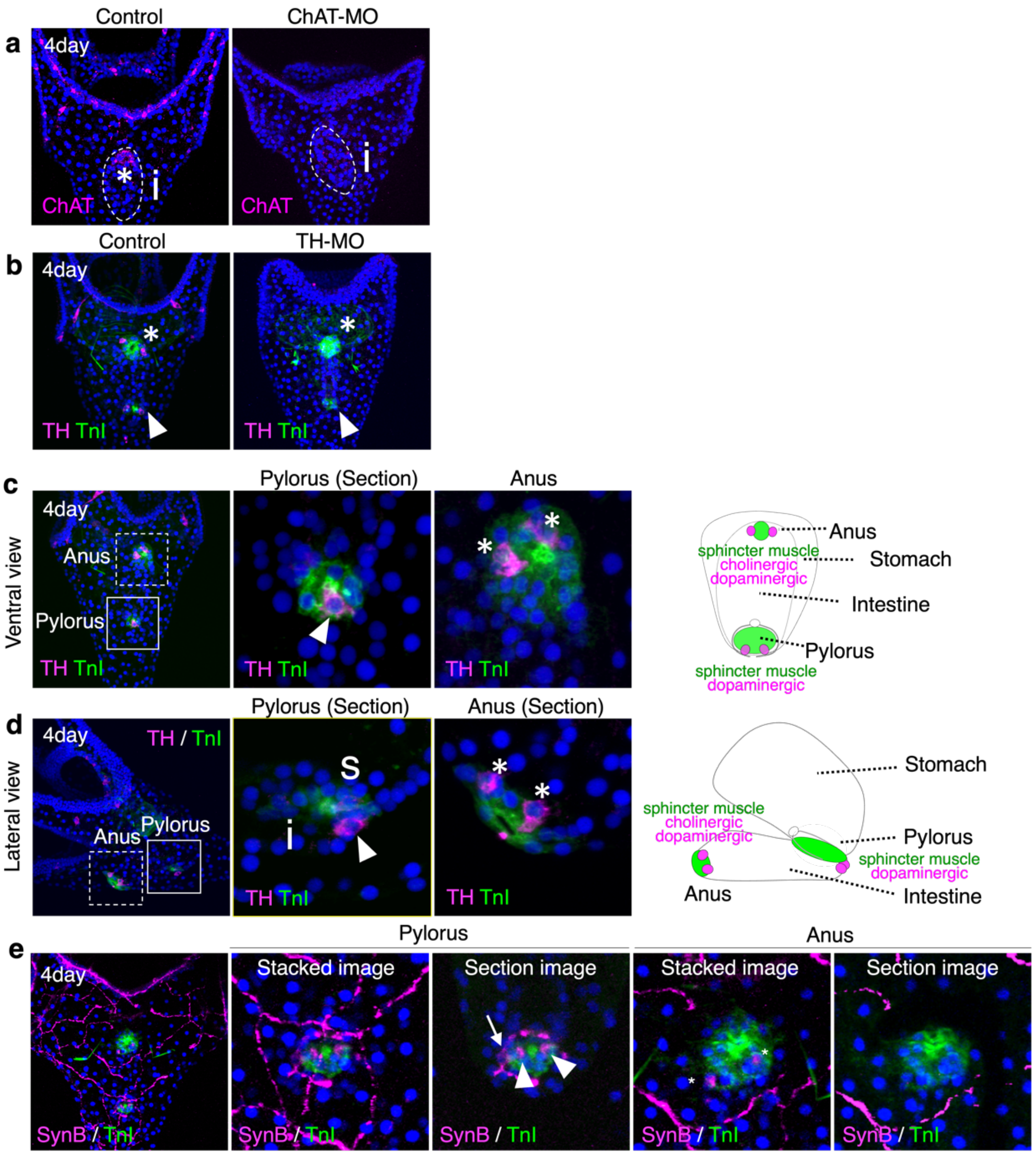

Details of Cholinergic and Dopaminergic Neurons in the Gut of Sea Urchin Larvae.

a. Anti-ChAT antibody signals accurately indicate cholinergic neurons, confirmed by the absence of these signals in ChAT morphants (ChAT-MO). **b.** Anti-TH antibody signals specifically mark dopamine neurons, validated by their absence in TH morphants (TH-MO). **c,d.** The relationship between neurons and sphincters is demonstrated, with both pyloric (arrowhead) and anal (asterisks) neurons being dopamine-positive as shown by

TH antibody staining. Sphincters are visualized via TnI expression, with neurons embedded within the sphincter region. Ventral (**c**) and lateral (**d**) views are provided. s, stomach; i, intestine. In the schematic image, magenta cells and green areas indicate dopamine neurons and sphincters, respectively. **e.** The relationship between axons extending from the ciliary band (indicated by pan-neural SynB) and the pylorus and anus is explored. Axons on the epidermis in the stacked image look like reaching to both pylorus and anus but not in the case in the pylorus judged by sectioning image, suggesting that signals to the pylorus from serotonin neurons may be transmitted via secretion within the coelomic cavity. Section image of the pylorus shows axons from sEN (arrow) and pyloric dopamine neurons (iEN, arrowheads). Axons near the anus suggest possible direct innervation, indicated by the asterisks marking anal neurons.

Supplementary Figure 5

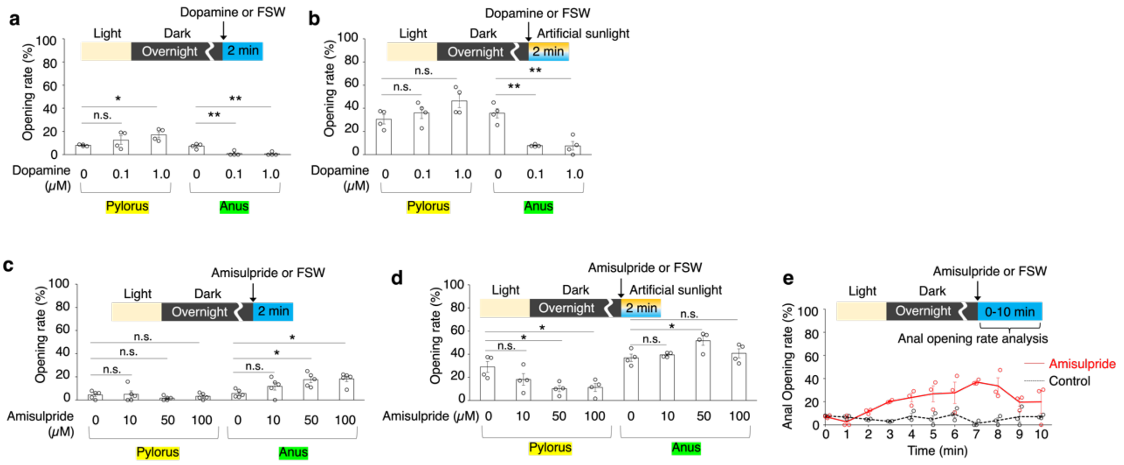

Role of the Dopamine Pathway in Regulating Pyloric and Anal Openings. a,b. Analysis of the opening rates of the pylorus and anus in sea urchins following treatment with varying concentrations of dopamine, assessed without (**a**) and with (**b**) photoirradiation. These results demonstrate the dopamine pathway's involvement in modulating opening behaviors under different light conditions (N [a] = 4 batches [each consisting of a different male and female pair], *n* [0 μM] = 47, 46, 55, 108 larvae, *n* [0.1 μM] = 27, 21, 21, 77 larvae, *n* [1 μM] = 19, 17, 22, 73 larvae; N [b] = 4 batches, *n* [0 μM] = 114, 72, 71, 94 larvae, *n* [0.1 μM] = 40, 29, 33, 53 larvae, *n* [1 μM] = 52, 24, 22, 30 larvae). **c,d.** Opening rates of the pylorus and anus post-treatment with the dopamine receptor inhibitor, amisulpride, at various concentrations, without (**c**) and with (**d**) photoirradiation (N [c] = 5 batches, n [0 μM] = 38, 67, 40, 50, 46 larvae, n [10 μM] = 33, 19, 29, 80, 52 larvae, n [50 μM] = 20, 55, 48, 61, 74 larvae, n [100 μM] =45, 40, 33, 47, 50 larvae; N [d] = 4 batches, n [0 μM] = 114, 72, 93, 40 larvae, n [10 μM] = 32, 17, 61, 29 larvae, n [50 μM] = 30, 19, 106, 40 larvae, n [100 μM] =43, 29, 61, 50 larvae). The data highlight the inhibitory effects of blocking dopamine receptors on opening responses. **e.** Time-course analysis of anal opening rates in sea urchins treated with or without

amisulpride (50 μM), in the absence of photoirradiation, providing insight into the temporal dynamics of dopamine-mediated regulation (N [control] = 3 batches, *n* [0 min] = 49, 55, 25 larvae, *n* [1 min] = 39, 20, 28 larvae, *n* [2 min] = 24, 21, 19 larvae, *n* [3 min] = 37, 30, 31 larvae, *n* [4 min] = 33, 25, 23 larvae, *n* [5 min] = 19, 30, 21 larvae, *n* [6 min] =48, 28, 22 larvae, *n* [7 min] = 28, 20, 14 larvae, *n* [8 min] = 25, 27, 13 larvae, *n* [9 min] = 30, 19, 19 larvae, *n* [10 min] = 23, 30, 17 larvae; N [amisulpride] = 3 batches, *n* [0 min] = 49, 55, 17 larvae, *n* [1 min] = 25, 15, 10 larvae, *n* [2 min] = 40, 39, 32 larvae, *n* [3 min] =36, 28, 10 larvae, *n* [4 min] =28, 24, 24 larvae, *n* [5 min] = 30, 23, 30 larvae, *n* [6 min] = 26, 10, 28 larvae, *n* [7 min] = 25, 23, 14 larvae, *n* [8 min] = 14, 19, 12 larvae, *n* [9 min] =22, 17, 16 larvae, *n* [10 min] = 33, 24, 10 larvae). We used one-way ANOVA followed by Tukey's post hoc test in (**a**-**d**). Statistical significance is denoted as $*p < 0.05$, $**p < 0.01$; n.s. = not significant. Error bars shown in all graphs indicate SEM. Source data are provided as a Source Data file.

Supplementary Figure 6

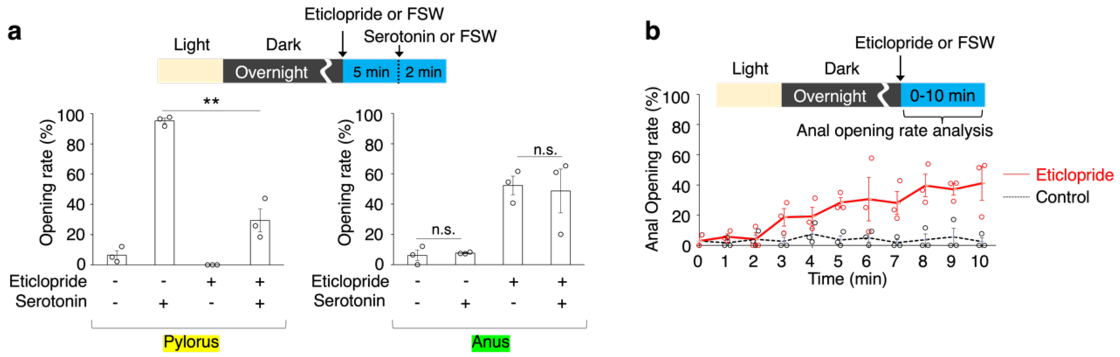

Interaction Between Dopamine and Serotonin Pathways in Regulating Sphincter Opening Behaviors. a. Comparative analysis of the opening rates of the pylorus and anus in larvae treated with a combination of a dopamine inhibitor and serotonin, illustrating the interplay between dopamine and serotonin pathways in modulating these behaviors (N = 3 batches [each consisting of a different male and female pair], *n* [eticlopride -, serotonin -] = 20, 47, 25 larvae, *n* [eticlopride -, serotonin +] = 24, 56, 41 larvae, *n* [eticlopride +, serotonin -] = 22, 47, 27 larvae, *n* [eticlopride +, serotonin +] = 35, 49, 41 larvae). **b.** Time-course analysis of the anal opening rates in sea urchins treated with or without the dopamine receptor antagonist eticlopride, in the absence of photoirradiation, shedding light on the temporal dynamics of dopamine's influence on opening behavior mediated by serotonin interaction (N [control] = 3 batches, *n* [0 min] = 55, 22, 57 larvae, *n* [1 min] = 28, 10, 19 larvae, *n* [2 min] = 35, 23, 21 larvae, *n* [3 min] =46, 18, 25 larvae, *n* [4 min] =43, 19, 20 larvae, *n* [5 min] = 48, 25, 22 larvae, *n* [6 min] = 45, 18, 32 larvae, *n* [7 min] = 37, 24, 35 larvae, *n* [8 min] =45, 28, 26 larvae, *n* [9 min] =50, 26, 35 larvae, *n* [10 min] = 37, 22, 26 larvae; N [eticlopride] = 3 batches, *n* [0 min] = 55, 22, 57 larvae, *n* [1 min] = 34, 21, 41 larvae, *n* [2 min] = 37, 25, 16 larvae, *n* [3 min] =49, 31, 46 larvae, *n* [4 min] =16, 13, 9 larvae, *n* [5 min] = 32, 16, 40 larvae, *n* [6 min] = 28, 11, 26 larvae, *n* [7 min] = 28, 17, 28 larvae, *n* [8 min] = 41, 21, 13 larvae, *n* [9 min] = 41, 22, 17 larvae, *n*

[10 min] = 53, 17, 37 larvae). Statistical significance is denoted as **$p < 0.01$; n.s. = not significant, Welch's t test (two-sided). Error bars shown in all graphs indicate SEM. Source data are provided as a Source Data file.

Supplementary Figure 7

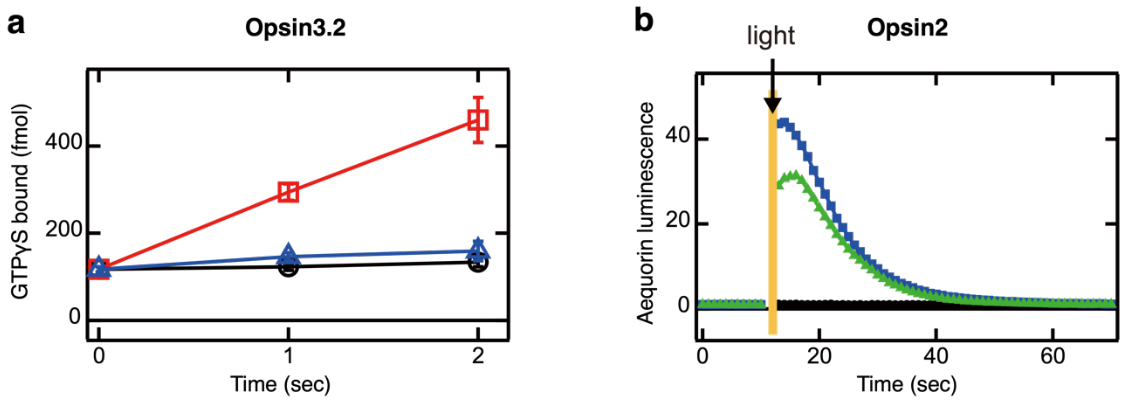

Activities of Opsin3.2 and Opsin2. a. Gi activation ability of Opsin3.2. The activation ability using the purified recombinant protein of Opsin3.2 was estimated by the GTPγS assay. The incorporation of GTPγS to Gi was measured in the dark (black circle), after blue light (460 nm) irradiation (red square) and after subsequent orange light (>580 nm) irradiation (blue triangle). Data were obtained at 0 ºC and are presented as the means ± SEM of three independent experiments. The coupling of Opsin3.2 with Gi is consistent with the previous observation of the orthologous opsin gene in amphioxus (*Branchiostoma belcheri*)[3]. **b.** Light-induced changes of intracellular Ca^{2+} level by Opsin2. The Ca^{2+} level in the Opsin2-transfected HEK293S cells was measured using aequorin-based luminescent assay. Luminescence change of aequorin was triggered by blue light (470 nm) (blue square) or green light (530 nm) (green triangle) irradiation for 5 sec. Luminescence change was also measured in the mock-transfected cells (black circle). Data are presented as the means of two independent experiments.

Supplementary Figure 8

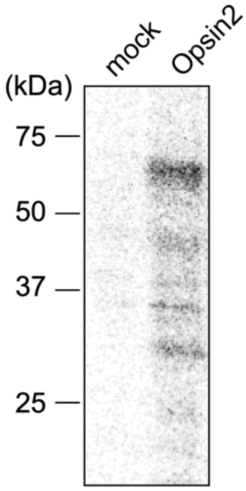

Western Blot Analysis for Opsin2 Expression in HEK293S cells. The expression of Opsin2 in HEK293S cells for the spectral measurement was confirmed by Western blot using Rho1D4 antibody. Extracts from Opsin2- or mock-transfected cells were subjected to SDS-PAGE, transferred onto a polyvinylidene difluoride membrane, and probed with Rho1D4. Immunoreactive proteins were detected using ECL (GE Healthcare) and visualized using a luminescent image analyzer (LAS 4000mini, GE. Healthcare) as previously described[4]. A major band around 65 kDa, which corresponds to the expected size of Opsin2, was detected only in Opsin2-transfected cells. Several lower molecular weight bands likely represent degraded protein products.

Supplementary References

1. Erickson, J. D., Schäfer, M. K. H., Bonner, T. I., Eiden, L. E. & Weihe, E. Distinct pharmacological properties and distribution in neurons and endocrine cells of two isoforms of the human vesicular monoamine transporter. *Proc. Natl. Acad. Sci. U. S. A.* **93**, 5166–5171 (1996).
2. Yaguchi, J. & Yaguchi, S. Sea urchin larvae utilize light for regulating the pyloric opening. *BMC Biol.* **19**, 1–14 (2021).
3. Tsukamoto, H., Terakita, A. & Shichida, Y. A rhodopsin exhibiting binding ability to agonist all-trans-retinal. *Proc. Natl. Acad. Sci. U. S. A.* **102**, 6303–6308 (2005).

4. Kojima, K. *et al.* Evolutionary steps involving counterion displacement in a tunicate opsin. *Proc. Natl. Acad. Sci. U. S. A.* **114**, 6028–6033 (2017).
