# Supplementary material for: Light-modulated neural control of sphincter regulation in the evolution of through-gut
Source: Nat Commun. 2024 Oct 18;15:8881. doi: 10.1038/s41467-024-53203-7 (PMC11489725; doi:10.1038/s41467-024-53203-7)
Supplement: Supplementary file 3 — Description of Additional Supplementary Files [file 41467_2024_53203_MOESM3_ESM.pdf]

Description of Additional Supplementary Files

File Name: Supplementary Movie 1
Description: . When the anus opens, the intestine contracts in sea urchin larva Lateral view.
